# Novel application of low pH-dependent fluorescent dyes to examine colitis

**DOI:** 10.1186/1471-230X-10-4

**Published:** 2010-01-15

**Authors:** Kazuhiro Ishiguro, Takafumi Ando, Osamu Watanabe, Hidemi Goto

**Affiliations:** 1 Molecular Biology and Pathogenesis of Gastroenterology, Nagoya University School of Medicine, 65 Tsurumai-cho, Showa-ku, Nagoya, Aichi 466-8550, Japan; 2 Department of Gastroenterology, Nagoya University Graduate School of Medicine, 65 Tsurumai-cho, Showa-ku, Nagoya, Aichi 466-8550, Japan

## Abstract

**Background:**

Endoscopy capable of fluorescence observation provides histological information on gastrointestinal lesions. We explored the novel application of low pH-dependent fluorescent dyes for fluorescence observation of crypt structure and inflammatory cell infiltration in the colon.

**Methods:**

Low pH-dependent fluorescent dyes were applied to the colonic mucosa of normal mice for observation under fluorescence stereomicroscopy system. We also examined mouse models of colitis, which were induced by trinitrobenzenesulfonic acid, dextran sulfate sodium or interleukin-10 deficiency.

**Results:**

Topical application of low pH-dependent fluorescent dyes revealed crypts as ring-shaped fluorescent stains by visualizing the mucin granules of goblet cells. Because of the minimal fluorescence intensity of the low pH-dependent fluorescent dyes in phosphate-buffered saline, it was not necessary to wash the mucosa before the fluorescence observation. 4-Nitro-7-piperazino-2,1,3-benzoxadiazole (NBD-PZ) was quicker to achieve complete staining (three minutes) than LysoSensor Green DND-153 and DND-189 (20 minutes). In each type of colitis, NBD-PZ revealed the destruction of the crypts as the disappearance of the ring-shaped fluorescent stains and the infiltration of inflammatory cells as the aggregation of punctate fluorescent stains through visualization of lysosomes.

**Conclusions:**

Low pH-dependent fluorescent dyes, especially NBD-PZ, are suitable for topical application to the colonic mucosa and have characteristics that allow for the histological examination of colitis.

## Background

Endoscopy is performed to diagnose and manage gastrointestinal diseases such as inflammatory bowel diseases [1]. Novel technologies for endoscopy provide diagnostic information that cannot be obtained with conventional endoscopy to accurately assess gastrointestinal lesions so that the appropriate medical treatment can be applied. Recent advances in endoscopy technologies have taken advantage of fluorescence, including confocal laser endoscopy [2-4] and autofluorescence-based endoscopy [5,6]. Autofluorescence-based endoscopy has its limitations. Autofluorescence images are not always accurate enough to discriminate between normal tissue, inflammation and neoplasia. The responsible fluorophores are not well defined apart from a few molecules such as collagen and reduced nicotinamide [5]. These limitations may be overcome by the application of fluorescent dyes, which visualize specific components in the mucosal layer, following autofluorescence observation.

Fluorescein is a fluorescent dye that is commonly used under confocal laser endoscopy. The intravenous administration of 100 mg/ml fluorescein enables clinicians to observe the distribution of fluorescein in the colonic mucosa and to acquire histological findings on neoplastic changes, thereby increasing the diagnostic yield and avoiding unnecessary biopsy [2]. The surveillance of neoplasms in ulcerative colitis has been improved by the combination of chromoscopy using methylene blue and confocal laser endoscopy using fluorescein [3]. Wang et al. reported the dynamics of fluorescein in the colonic mucosa, which reflect the functional behaviors of crypts, using a fibered confocal microscope with 5 mg/ml fluorescein following pretreatment with 5% acetic acid solution [4]. However, fluorescein exerts a high fluorescence intensity at the physiological pH (7.4-7.5) and is a nonspecific fluorescent agent that results in a homogenous staining of the mucosal layer, where the lamina propria in-between crypts is mainly visualized [5,7]. The merits of fluorescence observation will be further enhanced by the discovery of fluorescent dyes that have distinct characteristics from fluorescein.

Our previous study demonstrated that 4-nitro-7-piperazino-2,1,3-benzoxadiazole (NBD-PZ) is a low pH-dependent fluorescent dye and useful for the fluorescence observation of lysosomes as well as other low pH-dependent fluorescent dyes such as LysoSensor Green [8]. In the present study, we investigated the novel application of the low pH-dependent fluorescent dyes to the colonic mucosa and show that the characteristics of the low pH-dependent fluorescent dyes reveal the structure of crypts and the infiltration of inflammatory cells in the colon.

## Results

### Application of low pH-dependent fluorescent dyes to the mucosa of the normal colon

**Figure 1:**
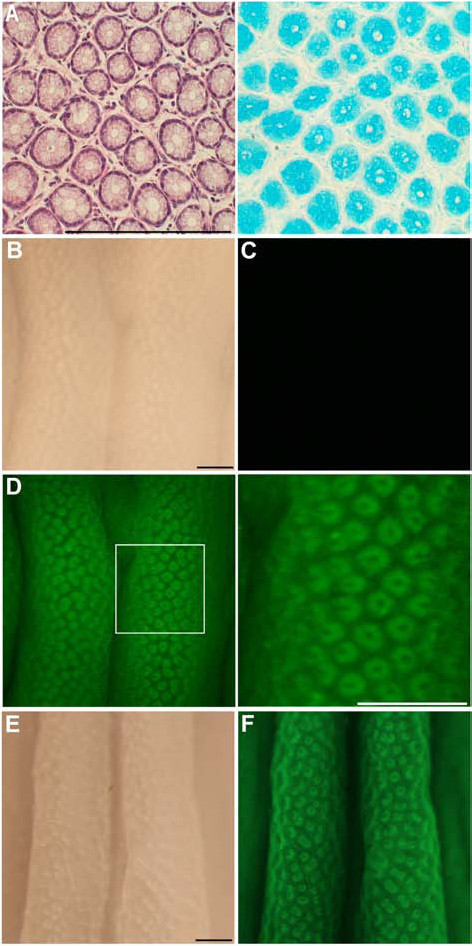
**Application of low pH-dependent fluorescent dyes to the mucosa of the normal colon**. (A) HE stain and Alcian blue (pH 2.5) stain of the colonic mucosa obtained from a normal BALB/c mouse. Bar, 200 μm. (B) Stereomicroscopic observation of the colonic mucosa obtained from the normal BALB/c mouse. Bar, 200 μm. (C) Fluorescence stereomicroscopic observation of the colonic mucosa in PBS only. (D) Fluorescence stereomicroscopic observation of the colonic mucosa in PBS containing 1 μg/ml NBD-PZ after incubation at 37°C for three minutes. A magnified image of the indicated area is shown in the right panel. We observed five samples and obtained similar findings. (E) Stereomicroscopic and (F) fluorescence stereomicroscopic observation of the colonic mucosa in PBS containing 1 μg/ml LysoSensor Green DND-189 after incubation at 37°C for 20 minutes.

Goblet cells are the major components of crypts in the colon of humans and rodents [9] (Fig. 1A, left panel). The mucin granules in goblet cells in the colon contain acid mucopolysaccharides [9], which are stained with Alcian blue (Fig. 1A, right panel), indicating that the intraluminal pH of the mucin granules is relatively low. Thus, crypts can be observed via visualization of the mucin granules with dyes exhibiting fluorescence dependent on a low pH, such as NBD-PZ [8].

To examine fluorescence observation with NBD-PZ, the colon was taken from BALB/c mice. The colon was opened longitudinally and pinned down on plates with the mucosal side up after the removal of stools. The mucosa was soaked in phosphate-buffered saline (PBS) (pH 7.4-7.5) alone or containing 1 μg/ml NBD-PZ, incubated at 37°C for three minutes, and observed under fluorescence stereomicroscopy system (excitation filter BP460-490, absorption filter BA510-550) (Olympus, Tokyo, Japan). We observed ring-shaped fluorescent stains in the colonic mucosa with NBD-PZ (Fig. 1B-D). Because of the minimal fluorescence intensity of NBD-PZ in PBS, it was not necessary to wash the colonic mucosa before the fluorescence observation. We also observed ring-shaped fluorescent stains in the colonic mucosa using other low pH-dependent fluorescent dyes, including LysoSensor Green DND-153 (data not shown) and DND-189 (Fig. 1E and 1F), although these dyes required 20 minutes of incubation to achieve complete staining. NBD-PZ is quicker to stain and less expensive than LysoSensor Green DND-153 and DND-189 [8]. Therefore, we used NBD-PZ in the subsequent experiments.

### Application of NBD-PZ to the mucosa of colitis

**Figure 2:**
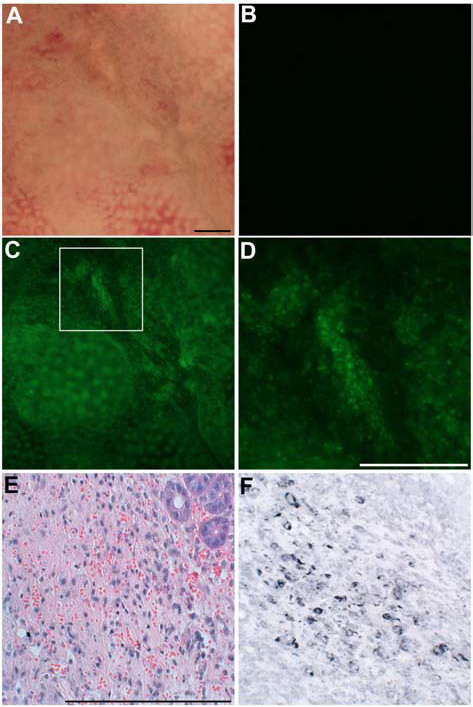
**Application of NBD-PZ to the mucosa of TNBS-induced colitis**. (A) Stereomicroscopic observation of the mucosa of TNBS-induced colitis. Bar, 200 μm. (B and C) Fluorescence stereomicroscopic observation of the mucosa of TNBS-induced colitis in PBS only (B) and in PBS containing 1 μg/ml NBD-PZ after incubation at 37°C for three minutes (C). (D) A magnified image of the indicated area in (C). We observed five samples and obtained similar findings. (E) HE stain of the colonic mucosa corresponding to (D). Bar, 200 μm. (F) Immunohistochemical stain of lysosomes using a section adjacent to the section shown in (E).

**Figure 3:**
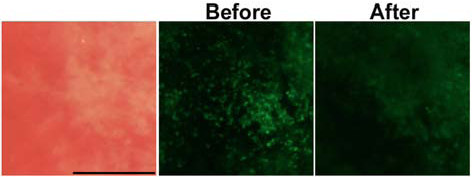
**The effect of bafilomycin A1 treatment**. The mucosa of TNBS-induced colitis was examined with 1 μg/ml NBD-PZ before and after 25 nM bafilomycin A1 treatment at 37°C for one hour.

To examine the fluorescence observation of colitis mucosa, BALB/c mice were intrarectally administered 100 μl of 50% ethanol containing 1 mg of trinitrobenzenesulfonic acid (TNBS). The enema of 1 mg of TNBS efficiently induces colitis and body weight reduction peaks at two days after the enema [10]. Therefore, the mice were sacrificed to remove the colon at two days after the enema. We applied NBD-PZ to the mucosa of TNBS-induced colitis as described above, and found that there was a disappearance of the ring-shaped fluorescent stains (Fig. 2A-D) that were observed in the normal colon (Fig. 1D). Histological examination showed that the disappearance of the ring-shaped fluorescent stains reflected the destruction of crypts and the loss of goblet cells in remnants of the crypts (Fig. 2E). Instead of the ring-shaped fluorescent stains, an aggregation of punctate fluorescent stains was observed (Fig. 2C and 2D). Certain mononuclear cells infiltrating into the mucosa contained lysosomes, which were detected by immunohistochemical staining with anti-lysosome associated membrane protein 1 antibody (Fig. 2E and 2F). The intensity of punctate fluorescent stains was substantially attenuated following treatment with bafilomycin A1, a vacuolar H^+^-ATPase inhibitor that increases lysosomal pH (Fig. 3). These findings indicate that topical application of NBD-PZ reveals the infiltration of mononuclear cells through the staining of their lysosomes, which can be visualized as the aggregation of punctate fluorescent stains.

**Figure 4:**
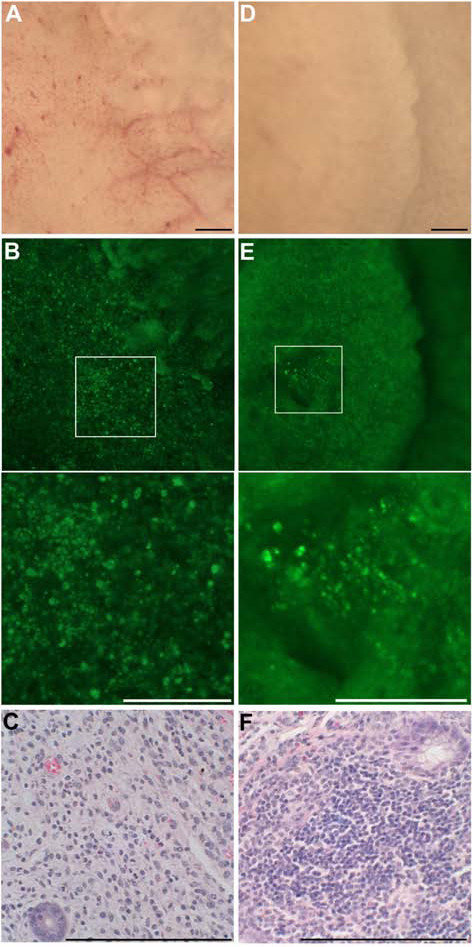
**Application of NBD-PZ to the mucosa of colitis induced by DSS or IL-10 deficiency**. (A) Stereomicroscopic and (B) fluorescence stereomicroscopic observation of the mucosa of DSS-induced colitis in PBS containing 1 μg/ml NBD-PZ after incubation at 37°C for three minutes. A magnified image of the indicated area is shown in the lower panel. Bar, 200 μm. We observed five samples and obtained similar findings. (C) HE stain of the colonic mucosa corresponding to the indicated area in (B). (D) Stereomicroscopic and (E) fluorescence stereomicroscopic observation with 1 μg/ml NBD-PZ of the mucosa of IL-10 deficiency-induced colitis. A magnified image of the indicated area is shown in the lower panel. (F) HE stain of the colonic mucosa corresponding to the indicated area in (E).

To apply NBD-PZ to other types of colitis, BALB/c mice received 3% dextran sulfate sodium (DSS) (36-50 kDa) in drinking water. The mice exhibited diarrhea 10 days later, after which experiment the mice were sacrificed to remove the colon. IL-10-deficient mice exhibited diarrhea at 3-4 months of age, after which time the mice were sacrificed to remove the colon. In DSS-induced colitis and in chronic colitis induced by IL-10 deficiency, we also observed a disappearance of the ring-shaped fluorescent stains and the aggregation of punctate fluorescent stains, which reflected the destruction of crypts and the infiltration of mononuclear cells, respectively (Fig. 4).

To compare findings of fluorescence observation and histological examination, IL-10-deficient mice at three months of age were sacrificed and the 1-cm length portion of the middle colon was removed. The colonic mucosa was observed with NBD-PZ under fluorescence stereomicroscopy system. Following the fluorescence observation, the colonic mucosa was fixed and histological examination was independently performed. We found statistically significant correlation between the disappearances of ring-shaped fluorescent stains and normal crypts (Table 1). Correlation was also found between the aggregation of punctate fluorescent stains and the infiltration of inflammatory cells (Table 2).

**Table 1:** Comparison of findings of crypt damage

Disappearance of ring-shaped fluorescent stains	Disappearance of normal crypts			
	not observed	observed		
		in < 1/3 area	in 1/3-2/3 area	in > 2/3 area
not observed	XXX			
in < 1/3 area		XX		
in 1/3-2/3 area		XX	XX	
in > 2/3 area			X	XXXX

Every X stands for one experiment performed. p = 0.0007 (Spearman's rank test).

**Table 2:** Comparison of findings of inflammatory cell infiltration

Aggregation of punctate fluorescent stains	Infiltration of inflammatory cells			
	
	not observed	observed		
		in < 1/3 area	in 1/3-2/3 area	in > 2/3 area
not observed	XXX			
in < 1/3 area		XXXX		
in 1/3-2/3 area			XXXXXXX	
in > 2/3 area				

Every ◦ stands for one experiment performed. p = 0.0003 (Spearman's rank test).

**Figure 5:**
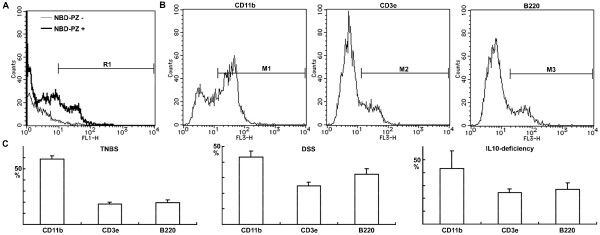
**Characterization of NBD-PZ-stained mononuclear cells isolated from the mucosa of colitis**. (A) Mononuclear cells were isolated from the mucosa of TNBS-induced colitis and incubated with 0.25 μg/ml NBD-PZ at 37°C for three minutes for flow cytometry. A total of 10,000 cells were analyzed. The cells in the R1 region were defined as NBD-PZ-positive. (B) Mononuclear cells were isolated from the mucosa of TNBS-induced colitis, incubated with 0.25 μg/ml NBD-PZ at 37°C for three minutes, and reacted with anti-CD11b, CD3e or B220 antibody on ice for 30 minutes. A total of 10,000 NBD-PZ-stained cells were analyzed by flow cytometry and the cells in the M1, M2 or M3 region were defined as CD11b-, CD3e- or B220-positive, respectively. (C) Mononuclear cells were isolated from the mucosa of colitis induced by TNBS, DSS or IL-10 deficiency to characterize the NBD-PZ-stained cells under flow cytometry. Data represent the percentages of CD11b-, CD3e- and B220-positive cells within the NBD-PZ-stained cell pool, respectively (means ± standard deviations, n = 3).

To characterize the cells stained with NBD-PZ, we isolated mononuclear cells from the mucosa of colitis for flow cytometry. The percentage of CD11b-positive cells was more than that of CD3-positive or B220-positive cells within the NBD-PZ-stained cell pool from each type of colitis (Fig. 5). We also observed that the majority of NBD-PZ-stained cells (55.2 ± 5.9%) expressed F4/80, another macrophage marker, using mononuclear cells isolated from the mucosa of TNBS-induced colitis. These findings suggest that macrophages are the primary population of NBD-PZ-stained mononuclear cells in colitis.

### Influence of NBD-PZ on the colonic mucosa and functions of the liver and kidney

**Figure 6:**
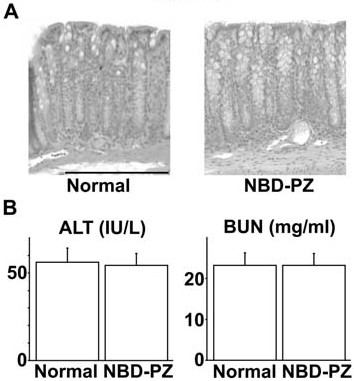
**Histological and biochemical examination of various organs following NBD-PZ enema**. (A) HE stain of the colon obtained from a BALB/c mouse at 24 hours after intrarectal administration of 100 μl PBS containing 2.5 μg/ml NBD-PZ. Bar, 200 μm. (B) ALT and BUN concentrations in the plasma obtained from BALB/c mice at 24 hours after the enema of 2.5 μg/ml NBD-PZ. Data represent the means ± standard deviations (n = 5).

**Figure 7:**
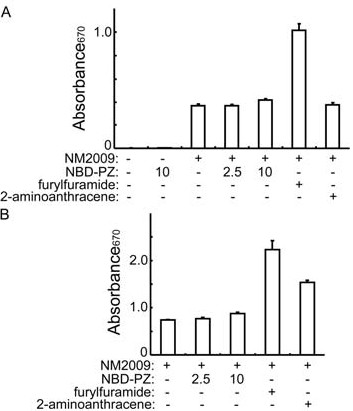
**Mutagenicity test of NBD-PZ**. (A and B) The *umu*-test bacterial strain, NM2009, was mixed with NBD-PZ (final 2.5 or 10 μg/ml), furylfuramide (0.03 μg/ml) or 2-aminoanthracene (0.3 μg/ml) in the absence (A) or presence (B) of liver homogenate, incubated at 37°C for two hours, and reacted with a chromogen. A compound is classified as mutagenic when it induces ≥ two times increase in absorbance at 670 nm compared with the sample that contains NM2009 only (the third or the first sample in A or B, respectively). Data represent the means ± standard deviations (n = 3).

We assessed the colonic mucosa by histological examination and measured the ALT and BUN concentrations in the plasma to assess liver and kidney functions at 24 hours after the intrarectal administration of 2.5 μg/ml NBD-PZ to mice. The enema of 2.5 μg/ml NBD-PZ did not damage the colonic mucosa (Fig.6A). Moreover, the enema affected neither ALT nor BUN concentrations in the plasma (Fig. 6B). We did not detect the mutagenicity of NBD-PZ at a concentration of 2.5 μg/ml (Fig. 7). These findings suggest that application of NBD-PZ to the colonic mucosa *in vivo *at concentrations up to 2.5 μg/ml may result in no overt damage to the colonic mucosa and functions of the liver and kidney.

## Discussion

The utility of fluorescence observation in the colon will be expanded by the application of fluorescent dyes that are capable of revealing the structure of the crypts and the infiltration of inflammatory cells. Within the crypts of the colonic mucosa reside goblet cells that have an apical accumulation of mucin granules [9]. In inflammatory diseases, macrophages play a critical role by linking the innate immune system with the adaptive immune system, and a previous report has indicated that activated macrophages have an increased number of lysosomes [11]. Both mucin granules and lysosomes are characterized by low intraluminal pH. Therefore, low pH-dependent fluorescent dyes are capable of revealing the structure of crypts and the infiltration of inflammatory cells, especially macrophages, by visualizing the mucin granules of the goblet cells and the lysosomes of the inflammatory cells, respectively.

NBD-PZ (excitation 470 nm, emission 540 nm) was originally designed to tag carboxylic acids in the presence of condensing agents such as diethyl phosphorocyanidate [12]. Our previous study demonstrated that the fluorescence intensity of NBD-PZ is increased below the physiological pH of 7.4-7.5 [8]. The fluorescence intensities of LysoSensor Green DND-153 (excitation 442 nm, emission 505 nm) and DND-189 (excitation 443 nm, emission 505 nm) are also elevated below pH 7.5 and below pH 5.2, respectively, according to the manufacturer's instructions. Topical application of these low pH-dependent fluorescent dyes enabled us to observe ring-shaped fluorescent stains, which reflected the distribution of goblet cells within the crypts of the colonic mucosa. Interestingly, NBD-PZ is quicker to achieve complete staining (three minutes) than LysoSensor Green DND-153 and DND-189 (20 minutes). The molecular sizes of NBD-PZ, LysoSensor Green DND-153 and DND-189 are 249, 356 and 398, respectively. The time required to achieve complete staining was not reduced by adjusting the molar concentrations of LysoSensor Green DND-153 and DND-189 up to the concentration of NBD-PZ (1 μg/ml = 4 μM) (data not shown). The small molecular size of NBD-PZ may be advantageous for its penetration into cells.

To observe the fluorescence images of the colitis mucosa with NBD-PZ, we used mouse models of acute colitis, which is induced by TNBS or DSS, and chronic colitis, which is induced in IL-10-deficient mice. The topical application of NBD-PZ revealed damage to the crypts that was accompanied by the loss of the goblet cells, which was visualized as the disappearance of ring-shaped fluorescent stains, and the infiltration of mononuclear cells through their lysosomes, which was visualized as the aggregation of punctate fluorescent stains. Although conventional colonoscopy can indicate the degree of inflammation in the colonic mucosa, there are sometimes discrepancies between colonoscopic and histological findings in patients with inflammatory bowel diseases at a clinically inactive stage, resulting in the difficulty of prognosis assessment [13,14]. Magnifying colonoscopy using 1 mg/ml methylene blue is useful to predict relapse in patients with quiescent ulcerative colitis by revealing pit patterns in the rectal mucosa, which reflect the arrangement of crypt orifices [15]. The mucosa of colitis is also examined histologically through fluorescence images with 1 μg/ml NBD-PZ reflecting both the crypt structure and inflammatory cell infiltration. The low fluorescence intensity of NBD-PZ in PBS avoids fluorescence background, which allows for the application of NBD-PZ to the colonic mucosa at need so that histological information can be collected from broad lesions. Therefore, fluorescence observation using NBD-PZ could become a useful technique for the histological evaluation of colitis and the assessment of prognosis in patients with inflammatory bowel diseases.

In the present study, we obtained fluorescence images with topical application of NBD-PZ under fluorescence stereomicroscopy system using mice. In the future, the clinical validity of NBD-PZ application must be assessed under endoscopy capable of fluorescence observation. Health damage induced by NBD-PZ has not been reported so far and NBD-PZ is not classified as a dangerous substance in its material safety data sheet. NBD-PZ does not affect the viability of the gastric cancer-derived cells, intestinal epithelium-derived cells or colon cancer-derived cells at concentrations up to 2.5 μg/ml (10 μM) even after 24 hours of incubation [8]. We did not find any adverse effects of NBD-PZ enema at concentrations up to 2.5 μg/ml in mice. Furthermore, NBD-PD did not show mutagenicity at a concentration of 2.5 μg/ml judging from *umu *gene expression, which is induced by genotoxins [16]. Although the expression of *umu *gene was slightly elevated with 10 μg/ml NBD-PZ, the elevation was much less than that with the positive control. However, the harmlessness of NBD-PZ should be further determined and careful monitoring should be performed at a clinical trial of NBD-PZ application to avoid any harmful events.

## Conclusions

Low pH-dependent fluorescent dyes, especially NBD-PZ, are suitable for topical application to the colonic mucosa to reveal the structure of the crypts and the infiltration of inflammatory cells by visualizing the mucin granules of the goblet cells and the lysosomes of the inflammatory cells, respectively. Although the harmlessness and the clinical validity of NBD-PZ remain to be determined, topical application of NBD-PZ would provide a novel technique that is able to histologically examine colitis under endoscopy capable of fluorescence observation.

## Methods

### Reagents

We obtained NBD-PZ from Tokyo Chemical Industry (Tokyo, Japan or TCI-America, Portland, OR), LysoSensor Green DND-153 and DND-189 from Molecular Probes (Eugene, OR). NBD-PZ was dissolved in dimethyl sulfoxide to a concentration of 2.5 mg/ml for the stock solution, which was stored at -20°C.

### Animal experiments

We purchased BALB/c mice from Japan SLC (Shizuoka, Japan) and IL-10-deficient mice from Jackson laboratory (Bar Harbor, Maine). All mice were treated according to the guidelines of the Institute for Laboratory Animal Research in Nagoya University School of Medicine and the approval of the ethics committee. To remove the colon, mice were sacrificed with cervical dislocation.

### Histological examination

The colon was fixed overnight in 4% paraformaldehyde and embedded in paraffin. After deparaffinizing the sections (6 μm thick), the samples were stained with hematoxylin and eosin (HE), or Alcian blue (pH 2.5).

### Flow cytometry

Mononuclear cells were isolated from the colonic mucosa as described previously [17]. The mononuclear cells were incubated with 0.25 μg/ml NBD-PZ at 37°C for three minutes, cooled on ice for five minutes, incubated with anti-Fcγ III/II receptor antibody (BD Biosciences), and then with PerCP-Cy5.5 anti-CD11b antibody (BD Biosciences), PE-Cy5 anti-CD3e antibody (eBioscience, San Diego, CA), PE-Cy5 anti-B220 antibody (eBioscience) or PE anti-F4/80 antibody (eBioscience) on ice for 30 minutes for flow cytometry.

### Biochemical measurements

Determiner ALT II (Kyowa, Tokyo, Japan) and Urea N B (Wako, Osaka, Japan) were used to measure alanine aminotransferase (ALT) and blood urea nitrogen (BUN) concentrations in the plasma, respectively.

### Mutagenicity test

We examined the mutagenicity of NBD-PZ with an *umu*-test kit (IWAKI, Tokyo, Japan), which determines *umu *gene expression induced by genotoxins [16], according to the manufacturer's instructions. Furylfuramide and 2-aminoanthracene were used as positive controls in the absence and presence of liver homogenate, respectively. Briefly, the *umu*-test bacterial strain, NM2009, was mixed with NBD-PZ (final 2.5 or 10 μg/ml), furylfuramide (0.03 μg/ml) or 2-aminoanthracene (0.3 μg/ml) in the absence or presence of rat liver homogenate, incubated at 37°C for two hours, and reacted with a chromogen. The expression of *umu *gene was assessed with absorbance at 670 nm. A compound is classified as mutagenic when it induces ≥ two times increase in absorbance at 670 nm compared with the sample that contains NM2009 only.

## List of abbreviations used

ALT: alanine aminotransferase; BUN: blood urea nitrogen; DSS: dextran sulfate sodium; HE: hematoxylin and eosin; NBD-PZ: 4-nitro-7-piperazino-2,1,3-benzoxadiazole; PBS: phosphate-buffered saline; TNBS: trinitrobenzenesulfonic acid.

## Competing interests

The authors declare that they have no competing interests.

## Authors' contributions

KI designed the study, directed all experiments and wrote the paper, TA performed fluorescence observation, and OW performed histological examination. HG contributed critically to the design of the study and the interpretation of data. All authors read and approved the final manuscript.

## Pre-publication history

The pre-publication history for this paper can be accessed here:

http://www.biomedcentral.com/1471-230X/10/4/prepub
